# Thermal Shock Resistance and Thermal Insulation Capability of Laser-Glazed Functionally Graded Lanthanum Magnesium Hexaluminate/Yttria-Stabilised Zirconia Thermal Barrier Coating

**DOI:** 10.3390/ma14143865

**Published:** 2021-07-10

**Authors:** Muhammed Anaz Khan, Annakodi Vivek Anand, Muthukannan Duraiselvam, Koppula Srinivas Rao, Ramachandra Arvind Singh, Subramanian Jayalakshmi

**Affiliations:** 1 Department of Mechanical Engineering, MLR Institute of Technology, Hyderabad 500043, India; muhammedanazkhan@gmail.com; 2 Department of Aeronautical Engineering, MLR Institute of Technology, Hyderabad 500043, India; vivekanandbit@gmail.com; 3 Department of Production Engineering, National Institute of Technology, Tiruchirappalli 620015, India; durai@nitt.edu; 4 Department of Computer Science and Engineering, MLR Institute of Technology, Hyderabad 500043, India; ksreenu2k@gmail.com; 5 Institute of Laser Optoelectronics and Intelligent Manufacturing, College of Mechanical and Electrical Engineering, Wenzhou University, Wenzhou 325035, China

**Keywords:** thermal barrier coating, yttria-stabilised zirconia (YSZ), lanthanum magnesium hexaluminate (LaMgAl_11_O_1__9_), thermal shock resistance, thermal insulation, laser glazing

## Abstract

In this work, functionally graded lanthanum magnesium hexaluminate (LaMgAl_11_O_19_)/yttria-stabilised zirconia (YSZ) thermal barrier coating (FG-TBC), in as-sprayed and laser-glazed conditions, were investigated for their thermal shock resistance and thermal insulation properties. Results were compared with those of a dual-layered coating of LaMgAl_11_O_19_ and YSZ (DC-TBC). Thermal shock tests at 1100 °C revealed that the as-sprayed FG-TBC had improved thermal stability, i.e., higher cycle lifetime than the as-sprayed DC-TBC due to its gradient architecture, which minimised stress concentration across its thickness. In contrast, DC-TBC spalled at the interface due to the difference in the coefficient of thermal expansion between the LaMgAl_11_O_19_ and YSZ layers. Laser glazing improved cycle lifetimes of both the types of coatings. Microstructural changes, mainly the formation of segmentation cracks in the laser-glazed surfaces, provided strain tolerance during thermal cycles. Infrared rapid heating of the coatings up to 1000 °C showed that the laser-glazed FG-TBC had better thermal insulation capability, as interlamellar pores entrapped gas and constrained heat transfer across its thickness. From the investigation, it is inferred that (i) FG-TBC has better thermal shock resistance and thermal insulation capability than DC-TBC and (ii) laser glazing can significantly enhance the overall thermal performance of the coatings. Laser-glazed FG-TBC provides the best heat management, and has good potential for applications that require effective heat management, such as in gas turbines.

## 1. Introduction

Thermal barrier coatings (TBC) are multi-layered ceramic coatings, usually used in gas turbines to impart thermal insulation to turbine components from hot combustion gases [1,2]. Typically, a TBC consists of two distinctive layers, namely (i) metallic bond coat and (ii) ceramic top coat. The metallic bond coat is coated over turbine components to provide better compliance with the ceramic top coat. The two layers of a TBC have distinct physical, thermal and mechanical properties. Thermal loading conditions are a major factor that determines the material selection for these two layers [3]. Turbine components such as combustor liners, blades, vanes and nozzles coated with TBCs are required to withstand high thermal loads and render thermal insulation, so as to achieve (i) higher engine efficiency, (ii) emission reduction, and (iii) cooling requirements. Myoung et al. [4] observed improvement in thermal durability upon air cooling thick ZrO_2_-8% Y_2_O_3_ TBCs coated on Ni-superalloy. The magnitude of thermal drop is influenced by factors such as heat transfer coefficients, heat flux, internal cooling, coating thickness and thermal conductivity. Ceramic top coats are expected to impart (a) low thermal conductivity, to enhance thermal insulation, (b) high strain tolerance under cyclic loading, to improve lifetime, and (c) stable microstructure, to minimise deleterious temperature effects such as phase transformations, grain growth and sintering. Yttria-stabilised zirconia (YSZ) is a widely used thermal barrier coating material. However, YSZ as a material has severe limitations, such as (i) ageing, (ii) post-sintering, and (iii) detrimental phase transformation (at temperatures >1200 °C) [5,6]. These limitations cause early failure of YSZ coatings. In YSZ, tetragonal to monoclinic phase transformation occurs during service and is the major reason for coating failure. Gu et al. [5] reported that YSZ-Y_3_Al_5_O_12_ (YAG) composite coatings can suppress monoclinic phase transformation. Freidrich et al. [6], in their work on YSZ, observed that above 1100 °C, the high oxygen ion conducting nature of zirconia caused increased diffusion of oxygen through the dense ceramic coating, resulting in oxidation of metallurgical interlayer. This consequently led to chipping of the ceramic coating, which limited its long-term high-temperature application [6]. To overcome the limitations of YSZ, (a) doping it with oxide stabilisers (e.g., MgO, Y_2_O_3_, Sc_2_O_3_, In_2_O_3_, CeO_2_, SnO_2_ and TiO_2_) has been investigated [7], and (b) other new materials such as those containing pyrochlore [8], fluorite [9], and perovskite [10] have been developed. Among the new materials, the hexaluminates (MMeAl_11_O_19_, M = La, Pr, Nd, Sm, Eu, Gd, Ca, Sr; Me = Mg, Mn, Fe, Co, Ni, Cu, Zn), which have a magnetoplumbite structure, exhibit improved structural and thermal stability up to 1400 °C. Hexaluminates have low thermal conductivity [6]. Among hexaluminates, lanthanum magnesium hexaluminate, LaMA (LaMgAl_11_O_19_) has good thermo-chemical stability [11], and also has an identical cyclic lifetime similar to that of YSZ [12]. The composition of LaMA is able to prevent post-sintering densification, as was reported by Freidrich et al. [6]. Additionally, high-temperature ageing in LaMA occurs more slower than other commercial zirconia-based TBCs, as was reported in [6]. This makes LaMA a promising material for TBC applications.

Conventional double-layer coatings are susceptible to cracking due to thermal stress mismatch and lower fracture toughness, which reduce their lifetime. Functionally graded thermal barrier coatings that have a multi-layered architecture are designed and developed with the aim of enhancing coating compliance and reducing thermal stress mismatch between the two layers, namely, the ceramic layer and metallic bond coat [13]. Functionally graded thermal barrier coatings have composite layers of two different ceramic materials. These coatings are designed such that their top layer is made from ceramic material that has a lower coefficient of thermal expansion, and its weight ratio with the other selected ceramic materials decreases in the subsequent underlying layers. As a consequence of such an architecture in functionally graded thermal barrier coatings, their physical and mechanical properties vary gradually across their coating thickness. Functionally graded thermal barrier coatings have improved thermal cycle lifetime and adhesion strength compared to conventional double-layer structures [12,14,15,16,17,18]. Kim et al. [15] investigated thermoelastic characteristics in TBCs with a graded layer between the top coat and bond coat. By using the finite element method (FEM), they identified that the functionally graded layer can considerably improve cycle lifetime. Kirbiyik et al. [16] synthesised multi-layered ceria and yttria stabilised zirconia (CYSZ)/Al_2_O_3_ ceramic TBCs, both in double-layered and functionally graded architectures. It was observed that the functionally graded architecture improved bonding strength between layers, and provided better thermal cycle performance than single-layered and double-layered coatings. Gok et al. [17] conducted thermal cycling experiments on multi-layered and functionally graded Gd_2_Zr_2_O_7_/CYSZ thermal barrier coatings. It was found that the functionally graded coating had lifetimes almost double those of the single-layered coatings.

Surface modification techniques have also been developed to increase the lifetime of TBCs. As an example, by optimising the coating parameters (such as material feed rate, spray distance, etc.), segmentation cracks can be induced in the top coat to provide better coating compliance [19]. However, in this case, the crack geometries become irregular and cannot be controlled during coating process. Several post-treatment processes have been developed to create segmentation cracks in the top coat. Laser glazing is one such advanced process, in which the top coat is remelted to a depth of few hundred microns by scanning a laser beam over the top coat. As a consequence, rapid resolidification within the treated depth induces a controlled network of segmentation cracks, in the direction perpendicular to the coating surface [20,21]. Glazing also reduces surface roughness [20,21]. Segmentation cracks are vertical cracks in the top coat, with a length at least half that of the thickness of the top coat [19]. The formation of segmentation cracks in a controlled manner relieves thermal stress (which is usually induced during thermal cycles) and improves coating compliance. Reduction in residual stress due to presence of segmentation cracks improves strain tolerance of coatings (i.e., accommodation of large thermal strains without failure [19]). In addition, they act as barrier for the propagation of delamination cracks (i.e., parallel cracks [19]). Segmentation cracks thus enhance thermal shock resistance [19,20,21,22,23,24]. Laser glazing thus improves the structural integrity of coatings [21,25,26,27]. Ghasemi et al. [27] laser glazed the top coat of YSZ-based nanostructured TBCs. They found that laser glazing eliminates surface porosities and reduces surface roughness of the coatings. Lee et al. [25] laser glazed plasma-sprayed CYSZ thermal barrier coatings. Their tests on thermal cyclic performance revealed a twofold increase in the lifetime of laser-glazed coatings when compared to their as-sprayed counterparts. The thermo-mechanical behaviour of laser-glazed TBCs is hence an important aspect that greatly influences the heat management performance of TBCs, and so requires detailed investigation.

The present investigation examines (i) the performance of two types of TBC architecture, namely, dual-layered and functionally graded architectures on the heat management capability of as-sprayed LaMgAl_11_O_19_/YSZ coatings. Thermal shock resistance and thermal insulation capability of the two types of archtiectures are evaluated to decipher the difference in their heat management capabilities; and (ii) the effect of laser glazing on the heat management capability of the coatings. The coatings were applied on Hastealloy (a nickel superalloy) surfaces.

## 2. Experimental Procedure

### 2.1. Test Substrate and Coating Materials

Hastelloy C-263 superalloy (Ni-Co-Cr-Mo alloy), used for combustion liners of gas turbines, was selected as the test substrate. The test coupons of 25 mm × 25 mm × 5 mm were machined and grit blasted (average surface roughness, R_a_~3 to 4 microns). Prior to coating, the coupons were degreased in an acetone bath. To synthesise LaMgAl_11_O_19_ (LaMA), a high-temperature solid-state reaction strategy was followed (Equation (1)). La_2_O_3_ oxide powder was preheated at 973 °C for 2 h, as it absorbs moisture and converts to lanthanum hydroxide [5,28]. Commercially available La_2_O_3_, Al_2_O_3_ and MgO were blended in a ball mill with 2:11:1 molar ratio.

2MgO + 11Al_2_O_3_ + La_2_O_3_ → 2LaMgAl_11_O_19_ (1)

Next, the blended powder was ball milled for 5 h and was subsequently heated in a ceramic tubular furnace at 1000 °C for 7 h. Heating temperature was progressively increased to 1650 °C, for 10 h. Eventually, the synthesised LaMA powder was ball milled and dried to obtain free-flowing powder (average particle size: 45–130 µm). Commercially available 8 wt.% YSZ was used for preparing the composite coatings.

### 2.2. Coating Architecture

**Figure 1:**
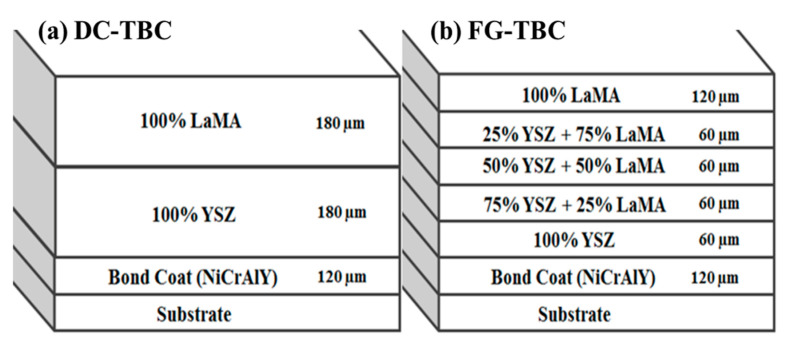
Coating architectures: (**a**) dual-layered architecture, DC-TBC and (**b**) functionally graded architecture, FG-TBC.

Two different coating architectures were followed: (i) double-layer structure (DC-TBC) having two separate layers of YSZ and LaMA above the bond coat and (ii) five ceramic layers above the bond coat with varying weight fraction of YSZ and LaMA (FG-TBC). Bond coat was made of NiCrAlY (composition: Ni-22Cr-10Al-1.0Y (wt.%)). Coatings were deposited via atmospheric plasma spray (APS) process (machine model: Sulzer Metco 9MP, Sulzer Metco India Limited). Optimised parameters of atomic spray process used to deposit the coatings are given in Table 1. The process parameters were selected based on initial trials and from information based on previous studies [29,30,31,32,33]. Total thickness of the deposited coatings was 480 µm. Architectures of DC-TBC and FG-TBC are shown as schematics in Figure 1.

### 2.3. Laser Glazing

DC-TBC and FG-TBC surfaces were laser glazed using a ytterbium-doped fibre laser (wavelength: 1080 nm). The laser was operated in continuous wave (CW) mode and the beam was kept at a slightly defocused position. Diameter of the circular laser beam was d_spot_ = 0.4 mm. Defocused position of laser was used so as to control the delivery of concentrated energy density and to eliminate the deterioration of ceramic layers upon interaction with the focussed beam. For this purpose, initial trials were conducted on single tracks on the developed coatings, with laser power setting at 500 W, 700 W and 900 W. Scanning speed was kept constant at 150 mm/min.

From post-deposition surface analysis, the optimal laser power setting was identified as 700 W and this laser power setting was used to glaze the coatings for thermal tests. The percentage of overlap was selected after measuring the glazed layer width of a single track. In the present work, the coated surfaces were glazed through 30% overlapped parallel tracks to achieve uniform remelting across the surface.

### 2.4. Surface Characterisation and Phase Analysis

Surface roughness of the TBCs was measured using a 3-D surface profilometer (Rtec instruments, San Jose, CA, USA) with vertical resolution less than 0.1 nm and lateral resolution of 100 nm. Presence of defects such as cracks and pores was identified using scanning electron microscope (SEM). Energy dispersive spectroscopy (EDS) was used for elemental analysis on the TBC surfaces. Phase analysis of the as-synthesised, as-sprayed and laser-glazed surfaces was conducted using X-ray diffraction (XRD, Rigaku ULTIMA-IV, Tokyo, Japan) with Cu-kα radiation. After the thermal shock resistance tests, the failure mechanism of TBCs were identified using SEM.

### 2.5. Thermal Shock Test

Thermal shock tests were conducted to determine the resistance of the coatings to thermal spalling, using a high-temperature muffle furnace at 1100 °C. Samples were heated for 10 min and were subseqeuntly quenched in water that was maintained at 20 to 25 °C. This heating–quenching cycle was repeated to determine the thermal shock resistance. Surfaces of the coated samples were monitored after every test, and the heating–quenching cycles were repeated until 20% of spallation was observed [34,35].

### 2.6. Infrared Rapid Heating Test

**Figure 2:**
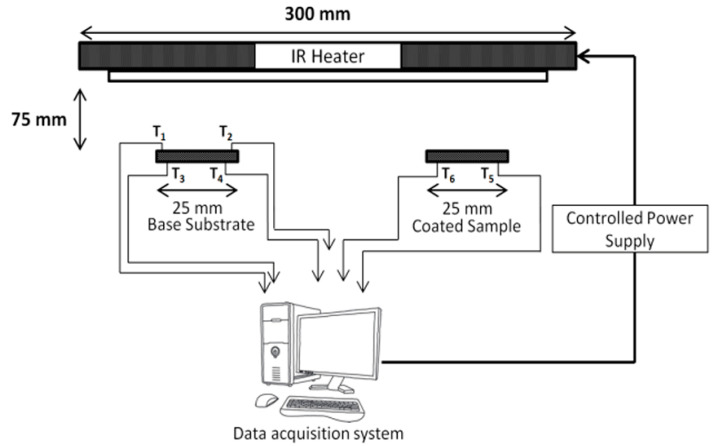
Schematic of infrared (IR) rapid heater.

Thermal insulation capability of the ceramic coated samples was evaluated using a 150 kW infrared (IR) rapid heater (Figure 2). Samples were mounted on sample holder, such that their coated surfaces were exposed towards IR heater. Ni-superalloy substrate was taken as the reference sample to characterise the thermal insulation of the coated test coupons. A gap of about 75 mm was maintained between the heater and the test coupons.

Type-R thermocouples were used to measure surface temperatures. Back wall temperature drop was measured with time. Thermocouples T_1_ and T_2_ were attached to the front side of the base reference sample facing the IR heater. Thermocouples T_3_ and T_4_ were attached on the back side of the base reference sample. T_3_ and T_4_ were the controller and redundant thermocouples. Thermocouples T_5_ and T_6_ were attached to the back side of the coated test coupon. Thermal insulation provided by ceramic layers in the coated samples was analysed by measuring the difference in temperature recorded by the thermocouples attached to the back side of the uncoated base reference sample and the coated test coupons. Test specimens were heated to 1000 °C at the rate of 25 °C/s. Peak temperature was attained in 100 s.

## 3. Results and Discussion

### 3.1. Surface Topography

The plasma spray process involves accelerating ceramic powders towards a target surface using high-energy plasma. In the case of TBCs, the size of the ceramic particles and surface roughness of bond coat influence their microstructure and surface roughness. During the APS process, molten and semi-molten particles impinge on the targeted substrate and/or previously deposited ceramic layers at higher temperature and pressure. This causes flattening and solidification of thin splats and results in the formation of anisotropic lamellar structure. The coatings consist of various types of defects, which include globular pores. Process parameters influence the adhesion strength of the APS coatings [36,37]. In the present work, the absence of microcracks between the layers indicates that the selected process parameters were optimal for producing good coatings.

**Figure 3:**
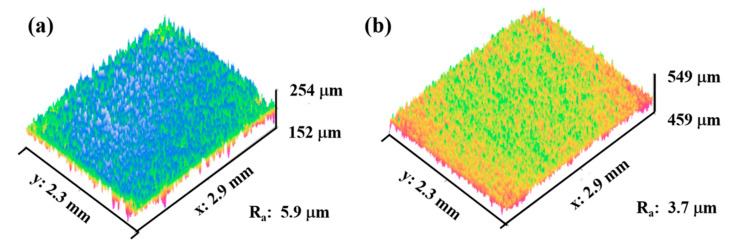
Three-dimensional surface topographies of FG-TBC: (**a**) as-sprayed surface and (**b**) laser-glazed surface. Laser glazing reduces roughness of ceramic coating.

Rougher bond coat surfaces facilitate better wettability of the molten splats and improve the adhesion of ceramic material to bond coat. This increases the coating lifetime [38,39]. During the spray process, as the molten splats solidify, the surface becomes rougher. The molten splats, as they reach the target surface, spread over the previously deposited solidified splats [22,23]. This imparts higher surface roughness to the as-sprayed coatings (Figure 3a). Laser glazing reduces the roughness of the ceramic coating (Figure 3b).

**Figure 4:**
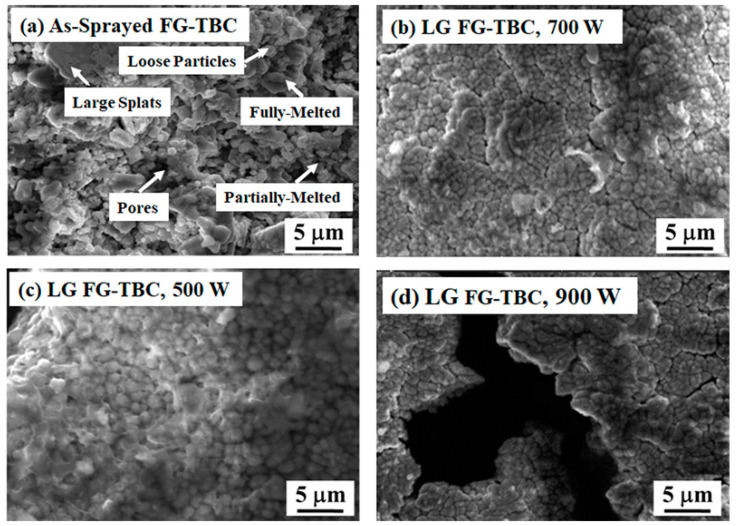
SEM images: (**a**) as-sprayed FG-TBC. Laser-glazed (LG) surfaces obtained using laser power setting of (**b**) 700 W, (**c**) 500 W and (**d**) 900 W.

Scanning electron microscopy (SEM) images of the as-sprayed and laser-glazed surfaces of FG-TBC are shown in Figure 4a–d. The as-sprayed FG-TBC surface shows partially and completely melted ceramic powders (Figure 4a). The as-sprayed FG-TBC surface is porous and contains micro-cracks (Figure 4a). During the spray process, the entrapped gas escapes through the molten ceramic, which creates bubbles and results in the formation of open pores over the surface of the as-sprayed coating. During the rapid solidification of the molten material, the induced thermal strain across the coating thickness and the relieving strain due to solidification cause micro-cracks in the as-sprayed coating [27]. However, the propagation of micro-cracks across the coating thickness is restricted by the mechanical interlocking of the overlapped resolidified splats. A significant difference between the surface topography of as-sprayed and laser-glazed surfaces can be observed (Figure 4a,b). Due to the laser glazing, the coarser and rougher surface of the as-sprayed ceramic is remelted and densifies (Figure 4b–d).

Laser glazing improves the surface characteristics of TBC by increasing microhardness, sealing surface porosity, reducing surface roughness, minimising the bending modulus of coatings, and by creating a controlled network of segmented cracks over the coatings [21,40,41]. The laser glazing parameters can be varied to obtain significant variation in surface morphology over the glazed surfaces [42]. Both pulsed wave and continuous lasers can be used for surface glazing. Important laser parameters include pulse power, peak power, pulse length, pulse shape, laser beam wavelength, laser scanning speed and the geometry of the laser beam (i.e., depth of focus, spot size) [43]. In the present work, the laser scanning speed was kept at 150 mm/min, which was selected on the basis of preliminary trials. Upon visual inspection of the laser-glazed surface, it was seen that the colour of the coatings changed from a pale grey to a light yellowish glossy surface. This change in colour is known to occur during laser glazing, and indicates optimal laser glazing conditions. Scanning speeds greater than 150 mm/min cause a higher thermal gradient across the coating thickness and a higher rate of thermal stress [44].

Upon interaction with the laser beam, the pores and micro-cracks heal significantly, leading to a homogeneously resolidified net-shaped structure (Figure 4b, laser glazed at 700 W). Segmentation micro-cracks occur due to the higher solidification rate imparted by the raster scanning of the laser source at the optimal laser power settings of 700 W. Segmentation cracks are known to influence thermal shock resistance and thermal cycle lifetime in TBCs [19,22,25,45,46,47]. The partially dense surface topography of FG-TBC glazed at 500 W (Figure 4c) indicates that this laser power level was not sufficient to glaze the surface effectively. The presence of macro-cracks on the surface of FG-TBC glazed at 900 W (Figure 4d) indicates that this laser power level was not optimal for laser glazing. Based on these observations, a laser power level of 700 W was selected to laser glaze the coatings for their investigation.

**Figure 5:**
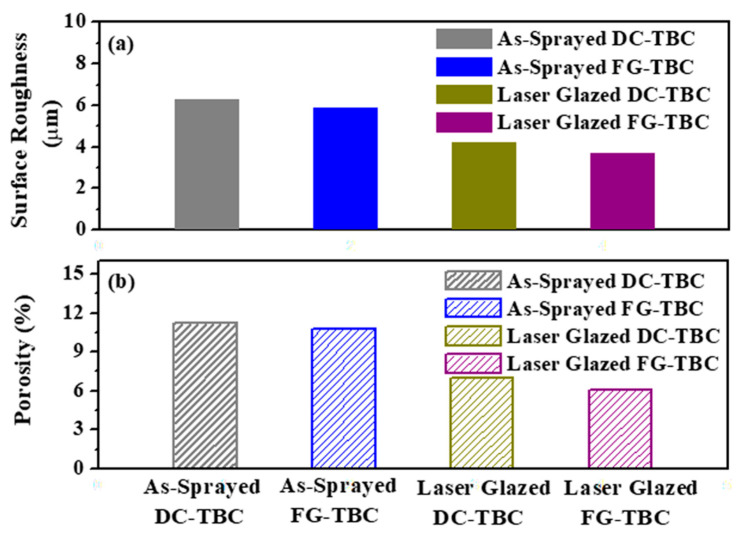
(**a**) Surface roughness (μm) and (**b**) surface porosity (%) of as-sprayed and laser-glazed coating surfaces.

**Figure 6:**
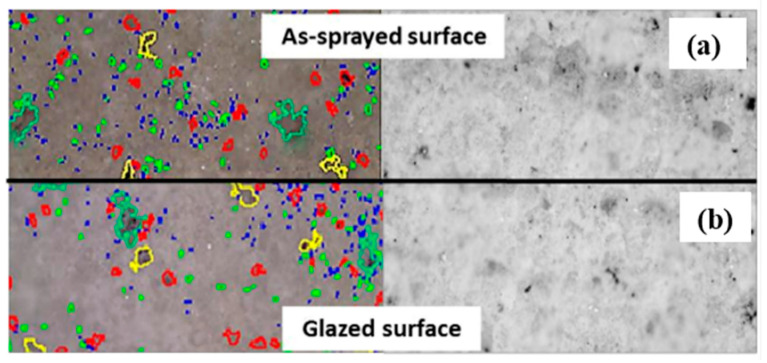
Optical microscopic images of surfaces of: (**a**) as-sprayed and (**b**) laser-glazed FG-TBC. In the right-hand side images, pores appear in grey/black colours. The left-hand side pictures are from the image analysis of the respective optical microscopic images, in which different colours represent pores of different size.

Figure 5a,b show the surface roughness and surface porosity of the as-sprayed and laser-glazed DC-TBC and FG-TBC, respectively. The DC-TBC surface has higher roughness compared to FG-TBC surface, both in the as-sprayed and laser-glazed conditions. For both coating architectures, the as-sprayed surfaces are rougher than the laser-glazed surfaces. Partially melted particles (Figure 4a) cause higher roughness (Figure 5a). DC-TBC and FG-TBC both have higher porosity levels in their as-sprayed conditions (Figure 5b). Porosity in the coatings appears in the form of open pores, interlamellar pores (i.e., interlamellar spaces between splats), and globular pores, causing coating failure [48,49]. Open pores permit diffusion of oxygen ions from flue gas into the metallic bond coat, causing oxide formation (thermally grown oxides, TGOs [49]). TGOs cause coating failure at the bond coat interface. The interlamellar pores that form due to rapid solidification lead to delamination of coating [48]. Globular pores, a result of improper filling of the coating material, stacking inconsistencies, incomplete contact between the splats, and the presence of unmelted particles, are initiation sites for coating failures [39]. Post-processing treatments can reduce surface porosity, as is evident from the low porosity levels in the laser-glazed TBCs (Figure 5b and Figure 6).

During laser glazing, owing to the rapid melting and resolidification, the open pores on surface close; in other words, they get patched. Additionally, remelting and resolidification densifies the coating material and induces segmentation cracks in the coating [19,49]. The glazed FG-TBC surface thus has a lower surface roughness (3.7 µm) and a lower porosity level (6.1%). Similar behaviour was reported by Ghasemi et al. [27] when nanostructured TBCs containing a YSZ ceramic top coat were laser glazed. A significant reduction in surface roughness after laser glazing was observed. They reported that the surface roughness (Ra) of the as-sprayed coating was 9.2 μm, which upon laser-glazing was reduced to 2.5 μm. Furthermore, they observed that the as-sprayed surface had cracks, voids and pores. Upon laser glazing, they observed an absence of defects, complete resolidification, and a dense microstructure with segmentation cracks [27].

### 3.2. Microstructure and Phase Analysis

**Figure 7:**
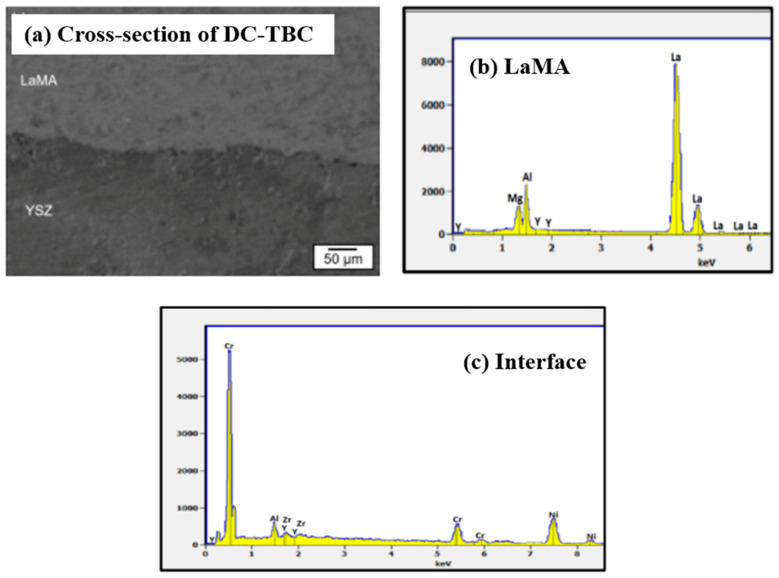
(**a**) SEM image of DC-TBC (cross-section). (**b**) EDS spectrum on LaMA. Peaks corresponding to La, Mg, Al and Y are present. (**c**) EDS spectrum of the YSZ/metallic bond coat interface. Peaks corresponding to Y, Zr, La, Al, Cr and Ni are present.

**Figure 8:**
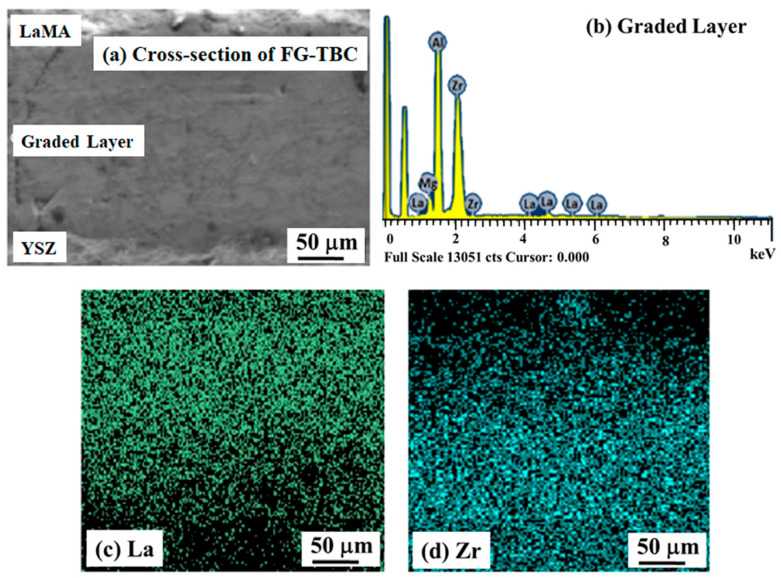
(**a**) SEM image of the cross-section of FG-TBC. (**b**) EDS spectrum across the graded layer showing the presence of La, Mg, Zr and Al elements. (**c**,**d**) Elemental maps showing the gradual variation of La and Zr across the coating thickness.

The interface between the YSZ and LaMA ceramic layers in the as-sprayed DC-TBC is shown in Figure 7a. Conformal deposition of LaMA over YSZ is evident. EDS analysis of the region (Figure 7a) confirms the presence of LaMA and YSZ elements (Figure 7b,c). The SEM cross-section image of FG-TBC and the corresponding EDS spectra are shown in Figure 8a,b. Elemental mapping of La and Zr taken across the FG-TBC (Figure 8c,d) shows gradual variation of the La and Zr elements across the coating thickness. This confirms the formation of the graded layer across FG-TBC.

**Figure 9:**
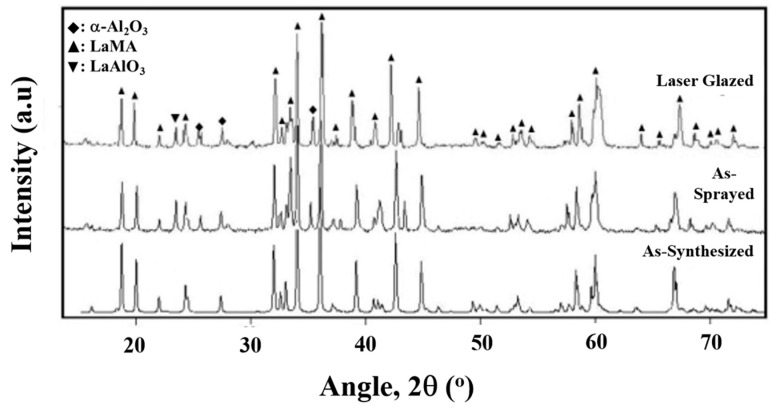
X-ray diffraction patterns of as-synthesised, as-sprayed and laser-glazed TBCs.

The XRD patterns for the as-synthesised LaMA powder, as-sprayed and laser-glazed surfaces are shown in Figure 9. Over the as-sprayed surface, the LaMA amorphous phase can be observed as a major phase with broader peaks. Peaks of LaAlO_3_ are present in the as-sprayed and laser-glazed samples due to partial decomposition of LaMA oxides during the spray process. Other volatile intermetallic peaks are not observed. Partial decomposition of LaMA oxides along with volatilisation during high-temperature synthesis reduced the percentage of volatile intermetallics [11]. For the laser-glazed surface, the narrow peaks indicate the crystallisation of LaMA oxides. The presence of α-Al_2_O_3_ peaks in the XRD pattern of the laser-glazed surface indicates the partial decomposition of LaMA oxides.

### 3.3. Thermal Shock Resistance

Thermal shock resistance is an important property of TBCs. The reliability of TBCs under extreme operating conditions is a critical factor. Gas turbine components operate with repeated run–stop cycles, inducing large fluctuations in temperature (i.e., cyclic thermal loads) to the TBCs used for the components. This causes thermal stresses across coatings [34,50,51,52,53]. As a consequence, degradation mechanisms manifest, such as sintering effect, thermal expansion, and high temperature friction. Evaluation of the thermal shock resistance of TBCs is therefore vital for their screening and selection for gas turbine components.

**Figure 10:**
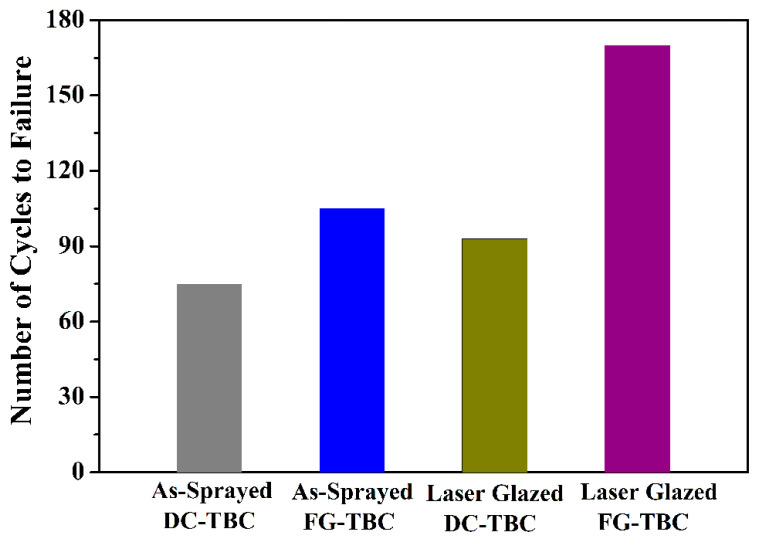
Thermal shock resistance, i.e., number of cycles to failure of DC-TBC and FG-TBC coatings in their as-sprayed and laser-glazed conditions.

The number of cycles to failure of DC-TBC and FG-TBC coatings are shown in Figure 10. In the as-sprayed condition, FG-TBC has a higher cycle lifetime than DC-TBC, by 30 cycles. In the laser-glazed condition, FG-TBC has a higher cycle lifetime than DC-TBC, by 77 cycles. Among the FG-TBC, the laser-glazed coating has a higher cycle lifetime than its as-sprayed counterpart, by 65 cycles. These results indicate that the laser-glazed FG-TBC has the best thermal shock resistance.

#### 3.3.1. As-Sprayed TBCs

Different failure mechanisms were observed for as-sprayed DC-TBC and FG-TBC. In the as-sprayed DC-TBC, horizontal cracks form and propagate at the interface between YSZ and LaMgAl_11_O_19_ layers due to the difference in their coefficient of thermal expansion (YSZ CTE: 10.2 × 10^−6^ K^−1^, room temperature to 877 °C; LaMgAl_11_O_19_ CTE: 5.13 × 10^−6^ K^−1^ [11]). Mismatch in the thermal expansion coefficient causes thermal stress mismatch at the interface, which changes the local volume along the interface. With the increase in the number of thermal cycles, spallation of the coating occurs due to the propagation of horizontal cracks.

**Figure 11:**
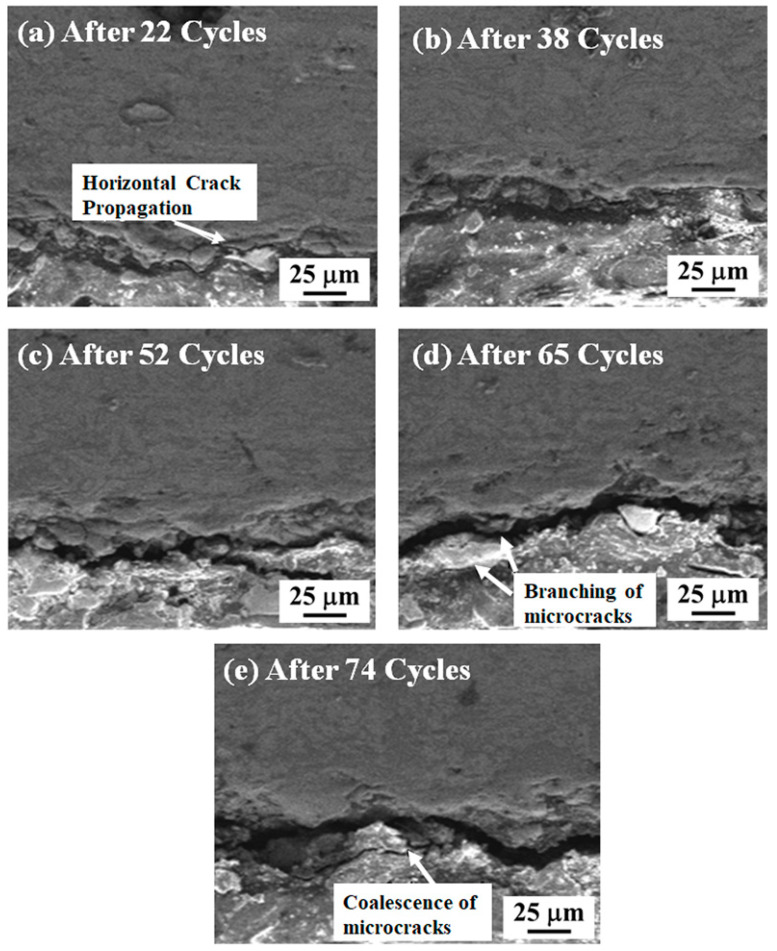
SEM images of as-sprayed DC-TBC after (**a**) 22, (**b**) 38, (**c**) 52, (**d**) 65 and (**e**) 74 heating–quenching cycles.

SEM images of as-sprayed DC-TBC, taken after different numbers of thermal cycles, are shown in Figure 11a–e. Horizontal cracks are initiated along the YSZ/LaMgAl_11_O_19_ interface after 22 cycles. The intensity of these cracks increases with increasing numbers of thermal cycles. The high thickness of both YSZ and LaMgAl_11_O_19_ (thickness: 180 µm each) induces a lower thermal gradient across the coating thickness and favours the accumulation of stress. Branching of the micro-cracks after 65 cycles can be observed in Figure 11d. The coalescence of the micro-cracks that can be seen in the sample after 74 cycles (Figure 11e) is caused by the accumulated thermal elastic strain, which is relieved rapidly upon quenching during the thermal shock test.

**Figure 12:**
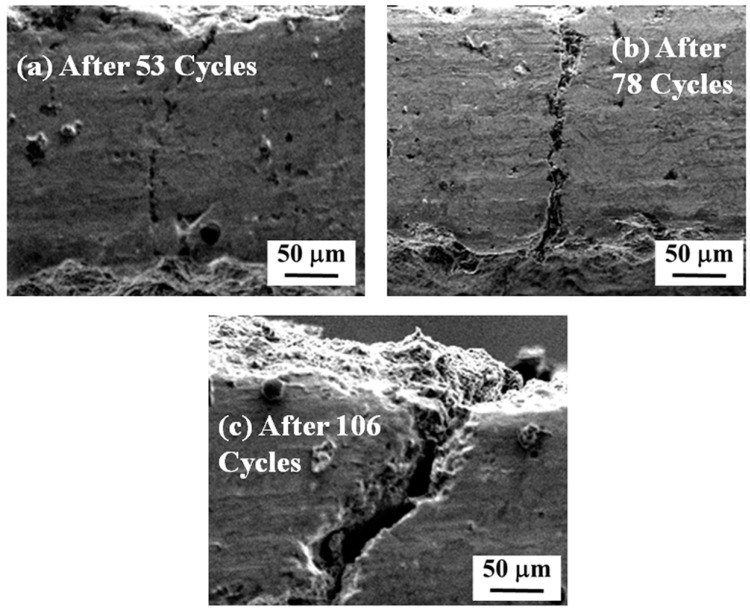
SEM images of as-sprayed FG-TBC after (**a**) 53, (**b**) 78 and (**c**) 106 cycles.

Compared to DC-TBC, the as-sprayed FG-TBC showed a different failure mechanism (Figure 12a–c). The graded layers of FG-TBC effectively prevent the accumulation of stress and provide better thermal insulation across the coating. The thermal insulation of the coatings is influenced by their microstructure and crystal structure [54]. FG-TBC has a top layer that consists of 100% LaMgAl_11_O_19_ (thickness: 120 µm), which has lower CTE, and its weight percentage decreases with subsequent underlying layers. The graded architecture reduces the propensity for the propagation of defects (such as micro-cracks) to the subsequent layers and to the substrate [55]. Due to this gradient architecture, stress accumulation in FG-TBC is lower than that in DC-TBC, which has a dual-layered architecture; consequently, FG-TBC has higher thermal shock resistance than DC-TBC (Figure 10).

In TBCs, there are two different crack propagation mechanisms: (i) cracks that propagate along the coated surface, i.e., parallel to the coated surface, termed inter-splat cracks; and (ii) cracks that are oriented across the thickness of coatings, i.e., perpendicular to the coated surface, termed intra-splat cracks [27,56]. Reports have shown that parallel cracks provide better thermal compliance and insulation than intra-splat cracks [57]. In the as-sprayed FG-TBC, the cracks initiate along the highly stressed brim region and propagate across the coating thickness. The intensity of these cracks increases with the number of thermal cycles (Figure 12). Coatings were partly purged along with the top coat and spalled within the ceramic layer.

#### 3.3.2. Laser-Glazed TBCs

Laser-glazed coatings have higher thermal shock resistance than their as-sprayed counterparts (Figure 10). Laser glazing causes the remelting and resolidfication of the coating surfaces and induces segmentation cracks. During laser glazing, the higher thermal gradients and non-uniform resolidification (i.e., rapid solidification ~10^7^ K/s) favour accumulation of thermal stress across the treated depth [20,46]. The shear force across the molten layer is accumulated due to the induced surface tension [20,47]. Gravitational force stabilises the induced shear force in the remelted zone. Therefore, the accumulated thermal stress to which segmentation cracks are subjected will be relatively lower than that experience by non-segmentation cracks. Thus, it can be observed that laser-glazed coatings have a higher cycle lifetime than the as-sprayed coatings (Figure 10). Similar observations have been reported previously, when plasma-sprayed ceria-yttira-stabilised TBCs were laser glazed [25]. The results showed that the thermal cycling lifetime of laser-glazed TBCs increased twofold [25]. Kadhim [33] treated surfaces of yttria partially stabilised zirconia (YPSZ) by laser sealing. Compared to the as-sprayed surface, laser surface processing effectively modified the surface layer by sealing the porosity and reducing surface roughness, and enhanced the thermal shock resistance due to the presence of segmentation cracks [33].

**Figure 13:**
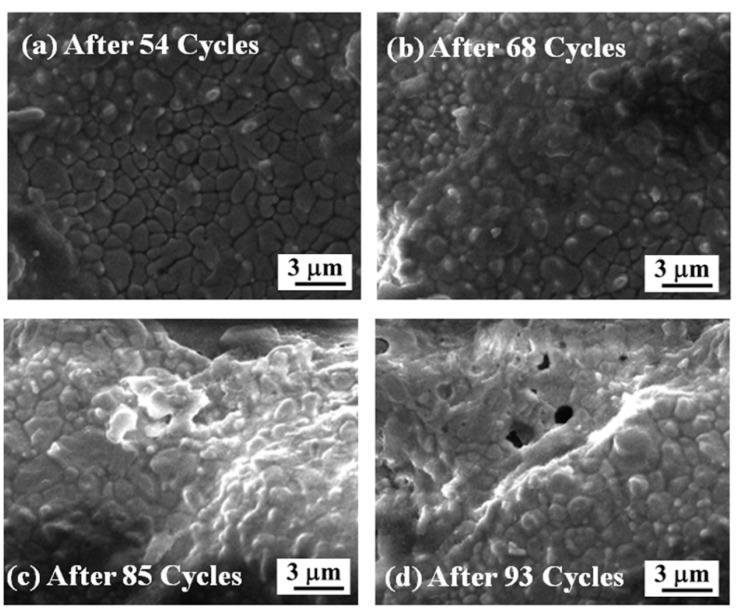
High-magnification SEM images of laser-glazed DC-TBC after (**a**) 54, (**b**) 68, (**c**) 85, and (**d**) 93 heating–quenching cycles.

SEM images of laser-glazed DC-TBC after 54, 68, 85 and 93 heating–quenching cycles are shown in Figure 13a–d. The segmentation cracks in the laser-glazed coatings accommodate thermal stresses and improve the strain tolerance. The difference in CTE between the dual layers induces thermal strain along the coating, causing formation and propagation of delamination cracks. The glazed coating surface becomes densified after 54 cycles (Figure 13), and the coating spalls. Guo et al. [23] reported significant improvement in thermal shock resistance of plasma-sprayed YSZ due to the presence of segmentation cracks. They observed that the coatings failed by spalling and delamination [23].

**Figure 14:**
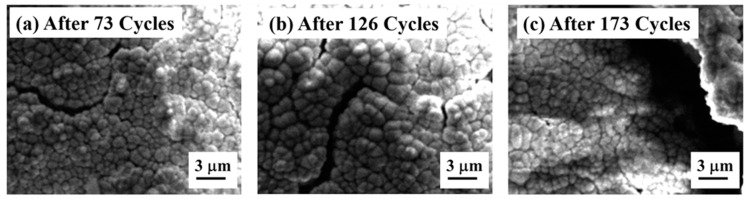
High-magnification SEM images of laser-glazed FG-TBC after (**a**) 73, (**b**) 126, and (**c**) 173 heating–quenching cycles.

Laser-glazed FG-TBC has a higher thermal shock resistance, i.e., a higher cycle lifetime, than its as-sprayed counterpart and laser-glazed DC-TBC (Figure 10). The better shock resistance of laser-glazed FG-TBC is due to (i) the gradient architecture and (ii) the beneficial effect of laser glazing. To elucidate, (i) gradient architecture prevents the accumulation of stress and reduces the crack propagation rate across the graded thickness of the coating [33]. In addition, it provides strain tolerance during heating–quenching cycles. (ii) Laser glazing densifies the structure, eliminates open pores, and thus prevents the diffusion of oxygen ions into the metallic bond coat. This reduces the propensity of oxide formation and coating failure thereof. In addition, the formation of segmentation cracks in the top coat upon laser glazing provides strain tolerance during heating–quenching cycles. With prolonged test cycles, the cracks propagated across the coating thickness and spalled the glazed layer, as shown in Figure 14a–c. The gradient ceramic layer is below the spalled region (not shown here).

### 3.4. Thermal Insulation Capability

**Figure 15:**
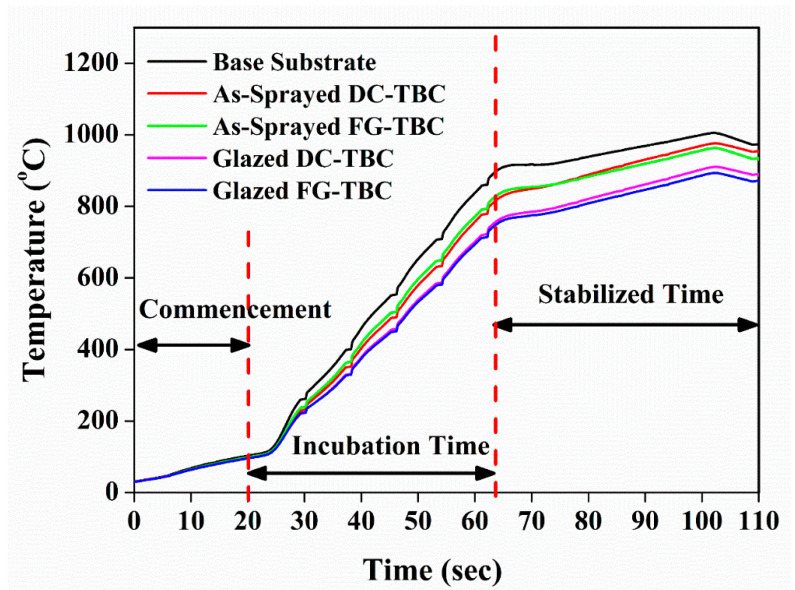
Back wall temperature plot of infrared rapid heating of DC-TBC and FG-TBC in their as-sprayed and laser-glazed conditions.

TBCs with both the types of architectures, in both as-sprayed and laser-glazed conditions, sustained the IR rapid heating test (1000 °C at 25 °C/s) without spallation. Acquired back wall temperature of the TBCs is shown in Figure 15. All traces have three distinct regions, namely, (i) the commencement region, wherein the temperature increases at a lower rate during the beginning of test (until 25 s), (ii) incubation time, wherein the temperature increases linearly (25 s to 65 s), and (iii) stabilised time, wherein the temperature reaches stable values. Two base reference samples (Hastealloy) were tested to study the accuracy of the data analyser in recording the drop in temperature. An identical back wall temperature of 998 °C was observed for both the samples. This means that the drop in the back wall temperature was 2 °C, which is considered to be negligible.

Back wall temperature drops of 67 °C and 83 °C were observed for the as-sprayed DC-TBC and FG-TBC coatings, respectively. Pores are known to impede thermal conduction and thereby enhance the thermal insulation of coatings [58,59]. In FG-TBC, which has multiple layers of YSZ-LaMA, mechanical interlocking of splats increases the roughness across the ceramic layers and imparts increased levels of porosity between the layers (i.e., interlamellar pores) [59]. Increased porosity induces increased thermal insulation. For this reason, FG-TBC has a higher back wall temperature drop. Reduction in the back wall temperature of the as-sprayed TBCs compared with the base reference material shows the thermal insulation capability of the ceramic coatings. Back wall temperature drops of 102 °C and 117 °C were observed for the laser-glazed DC-TBC and FG-TBC coatings, respectively. Under laser-glazed conditions, i.e., due to the densification of top coat, the interlamellar pores entrap gas and contribute towards enhancing thermal insulation [59]. Guo et al. [60] studied the effect of splat interfaces on the thermal conductivity of YSZ coatings, by finite element simulations and experiments. They identified that interlamellar pores cause lowering of thermal conductivity of TBCs, i.e., the interlamellar pores promoted thermal insulation. On similar lines, Wei et al. [61] conducted simulations and studied the effect of lamellar interspaces on thermal conductivity of TBCs. They opined that interlamellar pores trap gas molecules and limit the conduction of heat flow [60,61]. They reported that the presence of interlamellar pores can contribute up to 70% of reduction in thermal conductivity [61].

**Figure 16:**
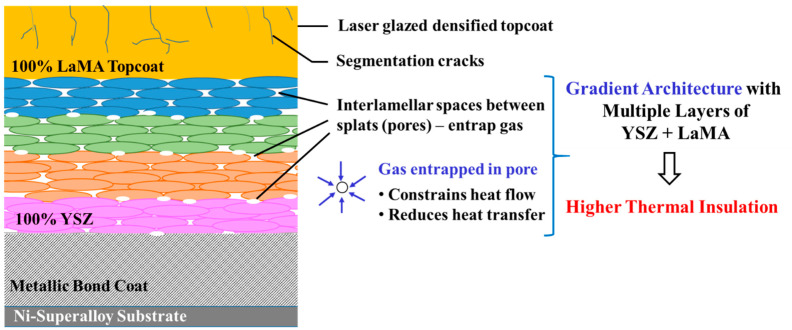
Schematic representation of the heat insulation mechanism in laser-glazed FG-TBC. Laser glazing densifies the top coat. Interlamellar space between splats (pores) entraps gas, constrains heat flow and prevents heat transfer across the coating thickness.

In the present case, laser-glazed FG-TBC showed better thermal insulation capability due to (i) increased formation of interlamellar pores owing to the multiple layers of YSZ-LaMA [5] and (ii) densification of the top coat, which leads to the entrapment of gas by interlamellar pores, preventing heat conduction across the coating thickness (schematically shown in Figure 16). Thus, by this mechanism, heat transfer is suppressed, and thermal insulation is enhanced in laser-glazed FG-TBC (Figure 15).

In thermal barrier coatings, thermal conduction is known to occur by phonon transmission. Ceramic oxides in thermal barrier coatings have lattice imperfections that scatter phonons [60]. Scattering of phonons hinders their free flow across coating thickness and consequently lowers thermal conductivity. In YSZ, the addition of yttria to zirconia requires O^2−^ vacancies in order to retain the electrical neutrality of ionic lattice. The O^2−^ vacancy and yttria scatter the incoming phonons across the lattice structure [27,60,62,63,64,65,66]. This phenomenon of phonon scattering induces thermal insulation, as there are insufficient free electrons (phonons are less effective in conducting heat energy compared to free electrons). YSZ and hexaluminate (LaMA) have low thermal conductivities (YSZ: 1.3 W/mK [66]; hexaluminates: 0.8 to 2.6 W/mK [6]). These ceramics, taken in combination to synthesise thermal barrier coatings with a functionally graded architecture, can provide good thermal insulation capability, as is evident from the results in the present investigation.

## 4. Conclusions

Lanthanum magnesium hexaluminate (LaMgAl_11_O_19_)/yttria-stabilised zirconia (YSZ) thermal barrier coatings were prepared with two architectures: (i) functionally graded coating (FG-TBC) and (ii) dual-layered coating (DC-TBC). The influence of the architecture type on the thermal shock resistance and thermal insulation capability of the TBCs was examined. TBCs were subjected to laser glazing, in order to determine the effect of glazing on their thermal shock resistance and thermal insulation capability. The main conclusions drawn from the investigation are as follows:

- Surface topography: Laser glazing significantly altered the surface topography of both coating architectures, such that the roughness and porosity of DC-TBC and FG-TBC on their surfaces was reduced. Densification of the top coat material due to laser glazing caused these reductions.
- Thermal shock resistance: FG-TBC has better thermal shock resistance, i.e., higher cycle lifetime, than DC-TBC, in both the as-sprayed and laser-glazed conditions.
  (a) As-sprayed DC-TBC spalled along the YSZ/LaMA interface due to thick dual layers and lower thermal gradient that caused stress accumulation along the interface. In as-sprayed FG-TBC, the functionally graded architecture reduced stress concentration, which increased cycle lifetime.
  (b) Laser-glazed FG-TBC has higher thermal shock resistance than laser-glazed DC-TBC due to (i) the formation of segmentation cracks, (ii) improved strain tolerance, and (iii) closure of surface pores.
- Thermal insulation capability: FG-TBC has better thermal insulation capability, i.e., higher back wall temperature drop, than DC-TBC, in both the as-sprayed and laser-glazed conditions. The multiple layers in FG-TBC cause increased formation of interlamellar pores. Laser-glazed FG-TBC showed better thermal insulation capability due to densification of the top coat, causing the entrapment of gas in interlamellar pores, which constrains heat transfer across the coating thickness.
- Laser-glazed FG-TBC has the best heat management, in terms of both thermal shock resistance and thermal insulation capability. It has good potential for applications that require effective heat management, such as in gas turbines.

## Figures and Tables

**Table 1:** Optimised parameters of atomic spray process used to deposit the coatings.

Coating Type	Current (A)	Voltage (V)	Stand-Off Distance (mm)	Primary Gas, Ar (slpm)	Secondary Gas, H_2_ (slpm)	Carrier Gas, Ar (slpm)
Bond Coat	550	75	110	35	14	2.3
Ceramic Top Coat	650	61	120	65	12	2.6

slpm: standard litres per minute.

## Data Availability

Data is contained within the article.
